# Data-to-text summarisation of patient records: Using computer-generated summaries to access patient histories^☆^

**DOI:** 10.1016/j.pec.2013.04.019

**Published:** 2013-08

**Authors:** Donia Scott, Catalina Hallett, Rachel Fettiplace

**Affiliations:** a University of Sussex, Department of Informatics, Brighton, UK; b SwiftKey Ltd., London, UK; c Eastbourne General Hospital, Eastbourne, UK

**Keywords:** Automatic summarisation, Electronic patient records, Natural language generation

## Abstract

**Objective:**

We assess the efficacy and utility of automatically generated textual summaries of patients’ medical histories at the point of care.

**Method:**

Twenty-one clinicians were presented with information about two cancer patients and asked to answer key questions. For each clinician, the information on one of the patients comprised their official hospital records, and for the other patient it comprised summaries that were computer-generated by a natural language generation system from data extracted from the official records. We measured the accuracy of the clinicians' responses to the questions, the time they took to complete them, and recorded their attitude to the computer-generated summaries.

**Results:**

Results showed no significant difference in the accuracy of responses to the computer-generated records over the official records, but a significant difference in the time taken to assess the patients' condition from the computer-generated records. Clinicians expressed a positive attitude towards the computer-generated records.

**Conclusion:**

AI-based computer-generated textual summaries of patient histories can be as accurate as, and more efficient than, human-produced patient records for clinicians seeking to accurately identify key information about a patients overall history.

**Practice implications:**

Computer-generated textual summaries of patient histories can contribute to the management of patients at the point-of-care.

## 1 Introduction

A patient's medical record typically consists of a range of documents, including test results, discharge reports, letters, observational notes and so on. These documents are often not available when and where they are needed, and even when they are, clinicians often do not have the time to read them carefully. Medical histories are also increasingly being captured as data in large repositories to serve administrative and research purposes. While such repositories hold information that is potentially valuable to clinicians, the information remains largely inaccessible to them since they have neither the expertise, time, nor inclination to extract what they need from the repository. We report on a presentation system designed to overcome some of these problems by generating tailored textual summaries of patients’ medical histories for use at the point of care.

There is a growing interest in representing patient histories as data rather than just text. In the UK, for example, this includes the NHS Spine that is part of Connecting for Health (a national initiative intended to facilitate clinical management), and the use for research purposes of large patient databases such as the General Practice Research Database (GRPD) [1] and The Health Improvement Network (THIN) [2]. The GRPD alone contains coded diagnostic, demographic and prescribing information for over 12.5 million patients from around 620 practices around the UK (approx. 7% of all UK practices). It has not gone unnoticed that one of the many advantages of representing clinical histories in this way is the ability to perform *in silica* experiments through statistical studies on data aggregated across patient populations. We explore here the possibilities for exploiting this data for yet another purpose: producing textual summaries of the history of individual patients automatically from the data. A facility such as this could mean that rather than having to wade through collections of paper documents that make up a typical “patient record”, or grapple with a complex database, practitioners would have at their disposal a new form of Electronic Patient Record that provides a customised view of a patient's history.

Current studies show that the quality of healthcare outcomes increases when doctors are able to spend sufficient time with patients to explore their symptoms, explain their condition and negotiate their treatment plan [3]. There is also agreement that the available duration of consultations in the UK is typically only between 7 and 10 min [4]. In most UK practices, clinicians are allocated 10 min for existing patients and 20 min for new patients. Since a consultation session also includes the time spent by the clinician familiarising herself with the patient's condition, the more time that is devoted to that activity, the less there will be for interacting with the patient.

Since clinicians have only a limited time for each patient, and since they clearly cannot be expected to be database experts, their ability to receive the information they require in an easily digestible form is obviously critical. A popular approach to this problem has been to build systems that produce *graphical* visualisations of the underlying patient data [5–10], but recent studies have shown that graphical visualisations of medical data are not always helpful for clinicians' decision-making [11,12].

Instead, we have chosen to explore the use of *textual* summaries, relying on the familiarity of this medium for presenting medical records. There are several reasons to believe that such summaries may be helpful to clinicians:

• Summaries provide a fast overview, and getting a fast overview is one of the four top reasons why clinicians read a patient's medical record [13]. One of the most important reasons for clinicians needing a fast overview is when the record concerns a patient who is unknown [14].
• Text is a natural format for clinicians; they read textual records as part of their day-to-day activities. Since text is a natural medium for presenting medical records, clinicians not need to be trained to read textual summaries, especially when they are written in the language of the genre (e.g., patients “present themselves” at an appointment, they “undergo” procedures, etc.). Indeed, there are some who argue that medical knowledge can only be fully expressed through natural languages [15].
• Natural language processing technology can provide useful tools to support clinical decision-making [16,17]. There have been several attempts that make use of natural language generation (NLG) to produce clinical summaries from data (e.g., [18,19]), but none have been subjected to quantitive evaluation.

We present here a computational system (a Report Generator) that automatically produces textual summaries of medical histories, and a study of its use by clinicians. We show that summaries, even when computer generated, can be a useful tool for clinicians at the point of care, providing an accurate overview of the patient's history in half the time.

## 2 Methods

### 2.1 The Report Generator

We developed a natural language generation system that produces a range of summarised reports of patient records from data-encoded views of patient histories derived from a repository of medical records of cancer patients, composed of narrative documents (e.g., letters, discharge reports, etc.) and structured data (e.g., test results, prescriptions, etc.) [20]. Although we are concentrating on cancer patients, we aim to produce good quality reports without the need to construct extensive domain models. Our typical user is a GP or clinician who uses electronic patient records at the point of care to familiarise themselves with a patient's medical history and current situation.

Information is extracted from medical narratives, using NLP techniques, as described in [21] and aggregated with structured data in order to build complex images of a patient's medical history which model the story of how the patient's illnesses and treatments unfolded through time: what happened, when, what was done, when it was done, and why. The resulting complex semantic network, termed by us a *Chronicle*, allows the construction of targeted summarised reports which do more than present individual events in a medical history: they present, in coherent text, events that are semantically and temporally linked to each other. We provide here a brief general overview; more detailed technical descriptions of the Report Generator are available in [22,23].

#### 2.1.1 Input

The input to the Report Generator is a Chronicle. The methodology involved in transforming an EPR into a Chronicle is complex and involves Information Extraction from narratives, solving multi-document coreference, temporal abstraction and inferencing over both structured and information extraction data [21]. The main advantage in using a Chronicle as opposed to a less structured Electronic Patient Record lies in the richness of information provided. Having access to not only facts, but to also the relations between them, has important implications in the design of the content selection and text structuring stages. This facilitates better and easier text generation and allows for a higher degree of flexibility of the generated text.

#### 2.1.2 Output

The output of the Report Generator is a range of textual summaries of the information contained in the Chronology. These range in length from short paragraphs to many pages. In the current implementation, the generator produces two main types of report. The first is a longitudinal report, which is intended to provide a quick historical overview of the patient's illness, whilst preserving the main events (such as diagnoses, investigations and interventions). It presents the events in the patient's history ordered chronologically and grouped according to type. In this type of report, events are fully described (i.e., an event description includes all the attributes of the event) and aggregation is minimal (events with common attributes are aggregated, but there is no aggregation through generalisation, for example).

The second type of report focusses on a given type of event in a patient's history, such as the history of diagnoses, interventions, investigations or drug prescription. This allows us to provide a range of reports that are presented from different perspectives. Under this category fall user-defined reports as well, where the user selects classes of interesting events (e.g., Investigations of type CT scan and Interventions of type surgery).

#### 2.1.3 Architecture

The system design of the Report Generator follows a classical NLG pipeline architecture, with a Content Selector, MicroPlanner and Syntactic Realiser [24]. These roughly correspond to deciding *what* to say, *how* to say it and then actually *saying* it. The MicroPlanner is tightly coupled with the Content Selector, since part of the document structure is already decided in the event selection phase. Aggregation is mostly conceptual rather than syntactic, therefore it is performed in the content planning stage as well.

**Fig. 1:**
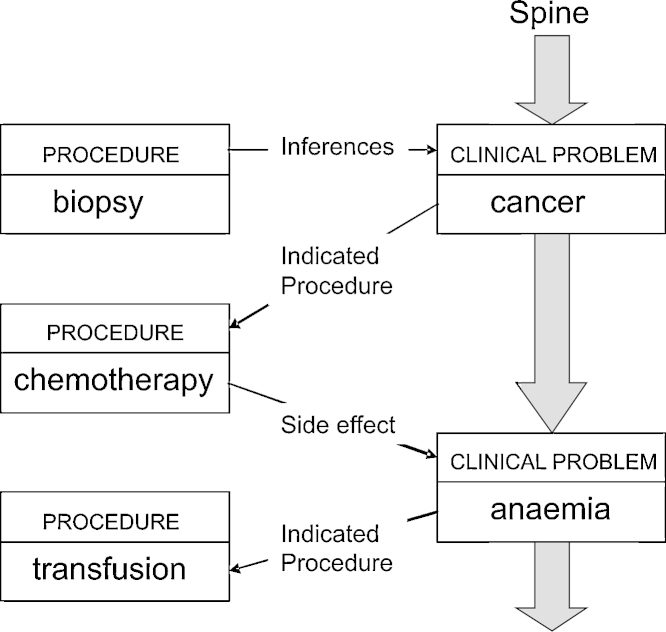
Result of content determination.

*Deciding what to say:* Starting from a knowledge base (the Chronicle) and the user's instructions (patient ID, time period, focus, etc.), the Content Selection module typically retrieves a semantic graph comprising a spine of focussed events elaborated by related events, as shown in Fig. 1. The events will have internal structure not shown in this diagram (e.g., the locus of the cancer and biopsy, the content of the transfusion, the dates of the biopsy and transfusion), represented formally as features on the event objects.

**Fig. 2:**
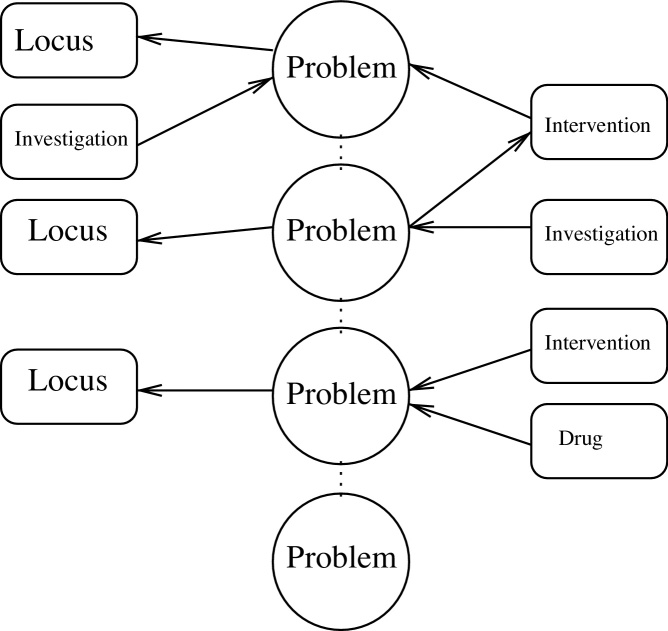
Example of a spine with a focus on Problems and a depth of 1.

The content selection takes into account the type and extent of the summary requested. For example, if a summary of the diagnosis is requested, the system will extract from the Chronicle only those events of type diagnostic (creating what we call the *spine* of a summary) and the events connected to events of type diagnostic up to a depth level indicated by the size of the summary (see Fig. 2). A depth of 0 will only list instance of diagnosis, a depth of 1 will also extract, for example, the consequence of a diagnosis (e.g., surgery), but no further events related to the surgery. The events extracted by this process will form the content of the summary (“what to say”).

**Fig. 3:**
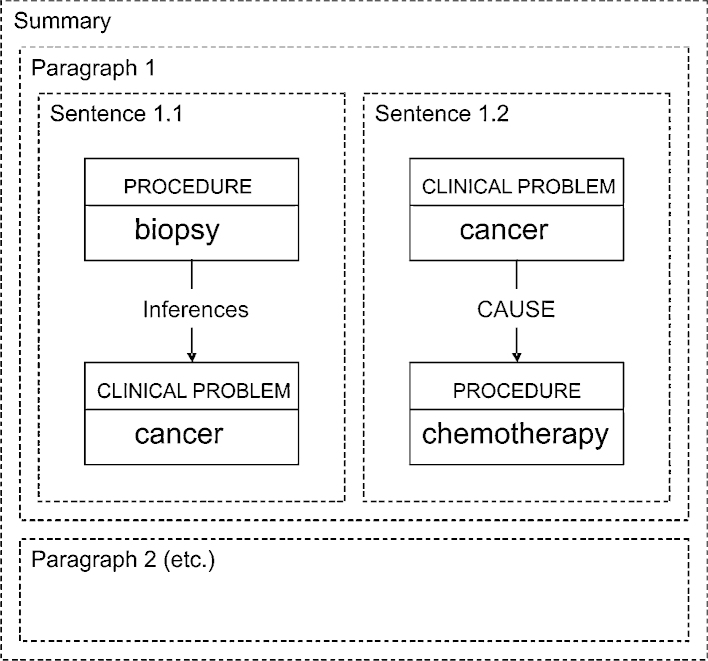
Result of microplanning.

*Deciding how to say it:* Starting from a spine-based semantic graph, a sequence of paragraphs is planned — usually, one for each event on the spine (along with the events elaborating it). Domain-specific relations are mapped to generic rhetorical relations, repeated events of a similar kind are aggregated, and the content is distributed among a set of sentences making up the paragraph; part of the result is shown by Fig. 3.

The Rhetorical Structure Theory [25,26] framework provides a well defined way of expressing discourse-level rhetorical relationships between utterances. The textual realisation of RST relations is not domain-specific, therefore the specific generation rules can be applied equally for the generation of medical summaries as well as any other type of English text. The RST framework is particularly suited to our specific application since the relations between chronicle events map naturally to RST schemas (e.g., we express facts such as inference (an event led to another) or causality (an event causes another)).

*Saying it:* Starting from a plan distributing the content among paragraphs and sentences, with some linking phrases and formatting already specified, a template-based grammar generates the surface forms of the sentences, producing as output a complete specification of the text. In our example, a template would map the domain-specific relationship

*inferences*(*biopsy*, *cancer*)

in Fig. 3 to the pattern

Patient had *Procedure* which revealed *ClinicalProblem*

while the generic rhetorical relation cause could be realised by the discourse connective ‘because of’. A possible output for the first paragraph would be as follows:

On 15th October, the patient had a biopsy of the left breast which revealed cancer. On 1st December, the patient started a chemotherapy course (CC1) because of the cancer.

The text generation system uses two types of grammar rules for realising the summaries. Firstly, a large standard generative grammar for English phrases and sentences, which consists of generic rules such as:

definite noun phrases = [definite article] + [determiner] + [noun] (for phrases)

or

causal relation = main clause + causal connector + subordinate clause (for sentences).

This helps generating constructs such as “*the clinical diagnosis*” or “*the patient underwent chemotherapy because of the cancer*”. These rules are static and independent of any new information available to the generation system, therefore no effort is involved in enhancing the rules when new data becomes available to the system.

The second set of generation rules are specific to the medical domain and more restricted in size. They govern the way the system expresses connections between words in the vocabulary, for example, the fact that the correct way of expressing an event of type surgical procedure is *“the patient underwent surgery”*. These rules are partially static in that they do not require re-writing or enhancing if we see new, unknown words which belong to a category known by the system (e.g., the fact that *“mastectomy”* is a brand new word of type surgical procedure doesn’t require rewriting the rules for surgical procedure. However, if the type of events in the Chronicle changes (e.g., if the system were to be applied to a new, non-medical, domain), we would need to manually create generation rules for each new type of event.

### 2.2 Evaluation

Can these automatically generated summaries perform a useful role in the clinical setting? We explored this question through a formal study with twenty-one clinicians at a teaching hospital. Of these 9 were final (5th) year medical students and 12 were qualified doctors with between 3 and 20 years of clinical practice. Their task was to consult two sets of clinical reports, each presenting the medical history of a cancer patient, and to answer ten questions about the patients’ condition. They were asked to perform this task in the context of a consultation they were about to have with a cancer patient who had been newly referred to them. Their task, then, was not to make a diagnosis or any other evaluation of the patient but to gather the important information that they would need before seeing the patient for the first time.

The two patients were randomly selected from the repository of clinical records of 22,500 deceased patients from the Royal Marsden Hospital in London. One (patient A) had a diagnosis of breast cancer (breast carcinoma with bony metastases); her hospital records cover 32 consultations over four and a half years, and consist of 43 documents; the other (patient B) had a diagnosis of invasive ductal carcinoma, with records covering 8 consultations over one year and consisting of 11 documents (see Tables 1 and 2). The records for each patient covers only the time they were treated at the Royal Marsden; patient A had received treatment elsewhere for five years prior and patient B for one year. Although already anonymised by the hospital, the records were subject to further careful scrutiny by two experts to remove all information that could identify the patient (e.g., occupation, consultant names, place names, etc.). Even so, all participants in our study were required to sign a non-disclosure agreement.

The ten questions addressed issues that our clinical partners advised were key ones for a clinician about to see new cancer patient:

• What is the presenting symptom/complaint?
• What was the stage of the cancer at diagnosis?
• What surgery was performed?
• When was chemotherapy started/ended?
• What was the first chemotherapy regimen given?
• What hormonal antagonists were given?
• When did the patient relapse?
• What was the site of the relapse?
• What was the last presenting complaint of the patient?
• What adverse effects to chemotherapy has this patient had?

Each clinician was presented with a set of records for each patient. For one patient they were given the original hospital records (consisting of a collection of documents); this mimicked the standard scenario for a doctor about to treat a new patient already diagnosed with cancer. For the other patient, they were given three summary records that were generated by the Report Generator: a full longitudinal summary, a summary from the perspective of clinical problems (e.g., cancer, anaemia or pain) and a summary from the perspective of curative procedures (e.g., chemotherapy, radiotherapy or surgery). Half of the subjects received the full records for Patient A and the summarised records for Patient B, and the other half received them the other way around. To avoid a biasing effect, half the subjects received the summaries before the full records, and the other half the other way around. All subjects received all questions in the same order.

The clinicians read the records or summaries (in different sessions) and then answered the 10 questions. For each set, they were given 5 min for a ‘preliminary reading’ before proceeding to the questions. They were told that they did not have to memorise the material and were allowed to refer to the documents throughout the question-answering session. The records and summaries were presented as paper documents, and the questions on a computer. The participants were not told that the summaries were automatically generated.

Each session started with a ‘dummy’ practice question to allow the user to become familiar with the question interface. Questions were presented one at a time on the computer screen and consisted of two parts that were presented on consecutive screens: a free-text box in which they could write their answers, followed by a multiple choice set of answers from which they had to choose one. They were able to proceed to the next question or question-part by clicking on a ‘Next’ button that appeared on the screen; they were told that it was important to perform this action immediately on answering the first part of each question as their responses were being timed, that they should select the same answer in the second (multiple-choice) part or, if it was not one of the given options, select “None of the above”; they were not allowed to return to the first part of any question to change their original answers. They could if they wished break between questions by clicking on an on-screen ‘Pause’ button.

At the end of the experiment, we asked the participating clinicians to complete a questionnaire aimed at capturing their general impressions of the utility of the generated summaries. When this was completed, we told them that the summaries were computer-generated by an AI-based natural language generation system whose input were facts presented in the hospital records. They all expressed surprise (and in some cases, bewilderment) that the summaries were not written by a human author.

## 3 Results

### 3.1 Results

We report here our finding with regard to the effect of the generated summaries (compared to the collection of documents that comprise the hospital records) on the *accuracy* of the assessments that the clinicians made on the histories of the individual patients and the *efficiency* of the clinicians in making their assessments.

#### 3.1.1 Accuracy

The results show that clinicians are slightly better at answering the set of key questions when using the automatically-generated record summaries than the (traditional) full records. They provide the correct answers 80% of the time when using the summaries, and only 75% of the time when using the full records (see Table 3). However, this difference is not significant (see Table 4). In other words, the use of generated summaries did not degrade the clinicians’ performance, even though record summaries are an entirely unfamiliar tool to them. Interestingly, there was no effect of level of experience (i.e., doctors vs students).

#### 3.1.2 Efficiency

The results show that use of the summaries reduced significantly the time taken to respond to the set of questions for each patient. Overall, using the summaries allowed the clinicians to shave off just over 50% of the time taken to answer all the questions compared to using the records (see Table 5). An analysis of variance on the data (see Table 6) shows a highly significant effect for the type of records, with summaries being a far more efficient tool than full records for providing answers to the set of key questions. Again, there was no effect of experience.

#### 3.1.3 Preference

At the end of the experiment, we asked the clinicians to answer a questionnaire aimed at their impressions of the utility of the summaries in the clinical setting, especially compared to the traditional records. Of the 21 clinicians, 19 completed the questionnaire.

We asked three forced choice questions:

• *Did you find the summaries helpful?*
• *If you had access to both the summaries and the narratives in a patient record, how would you make use of the summaries?*
• *How often would you use the summaries?*

The responses are shown in Tables 7–9 respectively.

We also asked them to answer the following questions in their own words: *Can you envisage contexts where you would use the summaries?* and *What things didn’t you like about the summaries?* Typical responses are shown in Tables 10 and 11 respectively:

### 3.2 Summary of results

An overwhelming majority of the clinicians reported that the generated summaries were very useful for answering questions about the patients’ condition. They said that, given the opportunity, they would make near constant use of the summaries, mostly by starting with the summaries and then using the records to double check information that they had located with the benefit of the summaries.

Clinicians reported a wide range of situations where they would wish to use summaries of the type shown to them in the study. This covered most clinical situations, but the most prevalent examples were ones where important decisions needed to be made in a short period of time, especially for unfamiliar patients (e.g., in Accident and Emergency (A&E) units, in outpatient clinics and for on-call doctors), for patients who were too confused or in too much pain to provide necessary information and for patients with very complex histories. Some clinicians also noted that the summaries would also help them carry out the more routine parts of their work – for example, they could be “cut and paste” into referral letters.

Although the participating clinicians found the summaries useful, the very fact that as summaries they are necessarily shorter, less detailed and incomplete means that they are not enough to rely on in general for making all clinical judgements. This is as expected.

An infrastructure that would allow summaries to be accessible at any time was seen by many to be very important. One of the clinicians also said that the legibility of the summaries was an added bonus, providing medico-legal robustness. She explained that:

“*We’re often criticised on the legibility of written notes and the failure of clinicians to clearly mark the patient's name, number and date of birth, plus the date and time seen on each medical incerpt, both because of coherence for anyone reading the notes but also, significantly, when litigation becomes involved. This, in turn, has potential financial implications for the hospital trust. The medico-legal body takes the approach that what is not documented or illegible did not happen until proven otherwise.*”

## 4 Discussion and conclusion

### 4.1 Discussion

Given the constraints of time that are imposed on medical staff, tools to provide quick and accurate information in an easily accessible form could prove useful. However, computerised aids are not always readily accepted by medical staff [27–29]. We have shown that NLG technology can indeed be employed successfully in a medical setting to produce compact, targetted textual summaries of a patient's history. In particular, we show that such summaries of large medical datasets can significantly improve the efficiency of clinicians in certain critical settings. Moreover, the clinicians in our study were overwhelmingly enthusiastic about the automatically generated summaries, a finding that is particularly encouraging given the novelty of the documents and the natural reluctance of clinicians towards computer-generated reports. The familiarity of the textual medium no doubt played an important role in the success of our system. Combined with graphical facilities, we suspect that it may be possible to increase even further the efficiency of clinicians in the specific context of making an initial assessment of a patient based solely on their medical history, and we are now investigating this.

Although the study reported here focuses on cancer treatment, the techniques that underpin the Report Generator can be applied to almost any medical context. Nevertheless, the Report Generator is to-date a proof-of-concept research system; transformation to a full-deployable clinical tool would require further software development and testing. Additionally, as with any data-presentation system, the accuracy of the generated summary is fully dependent on the accuracy of its input, in this case:

*Data quality* : the accuracy of the data contained in the patient record;
*Information extraction* : the accuracy of the process for extracting relevant information from the record, whether this process is carried out by AI-based information extraction systems or by suitably trained people.

In the language of AI, this is termed “*garbage-in, garbage-out*”.

### 4.2 Conclusions

This study demonstrates that AI technology can be successfully employed to write textual summaries of a patient's medical history. Such summaries are not only accurate (to the extent that the recorded patient data is accurate), but can provide clinicians with key information about a patient's history in about half the time that it would take if the clinician were instead having to search through the patient's textual record.

### 4.3 Practice implications

A significant portion of a clinician's time is taken up with non-clinical tasks such as reading the medical records of patients that they are about to see, or having seen the patient, writing letters or reports about the patient. Automatically generated summary overviews of a patient's medical history can potentially enhance doctor–patient interactions by significantly reducing the time required for doctors to carry out some of these tasks.

## Conflict of interest

The authors have no conflict of interest to declare.

## Figures and Tables

**Table 1:** Length of records for Patient A

	Full record	Summaries	
		Curative procedures	Clinical problems
Pages	54	2	2
Words	8190	299	310

**Table 2:** Length of records for Patient B

	Full record	Summaries	
		Curative procedures	Clinical problems
Pages	11	1	1
Words	3182	192	197

**Table 3:** Mean accuracy per set (mean number of correct answers)

	Full	Summary	Total
Student	7.78	8.00	7.89
Doctor	7.33	8.08	7.71

Total	7.56	8.04	

**Table 4:** Anova results for accuracy

Source	SS	df	MS	*F*	*P*
Subject type	0.335	1	0.335	0.295	0.5900
Record type	2.431	1	2.431	2.141	0.1516
rxc	0.716	1	0.716	0.631	0.4319
Error	43.139	38	1.135		

**Table 5:** Mean time per set (min)

	Full	Summary	Total
Student	12.23	6.21	9.22
Doctor	11.58	5.92	8.75

Total	11.90	6.07	

**Table 6:** Anova results for efficiency

Source	SS	df	MS	*F*	*P*
Subject type	81589792	1	81589792	0.168	0.6842
Report type	1.26E+10	1	1.26E+10	25.976	≤.0001
rxc	12188712	1	12188712	0.025	0.8750
Error	1.85E+10	38	4.86E+08		

**Table 7:** Did you find the summaries helpful?

Score	Number of clinicians
1 (not helpful at all)	0
2	0
3	1 (5%)
4	10 (53%)
5 (very helpful)	8 (42%)

**Table 8:** If you had access to both the summaries and the narratives in a patient record, how would you make use of the summaries?

Score	Number of clinicians
On their own	2 (10%)
Look up some information in the record and some in the summaries	3 (16%)
Use the summaries to locate information and records to double check	14 (74%)
Use the records to locate information and summaries to double check	0
Wouldn’t use the summaries at all	0

**Table 9:** How often would you use the summaries?

**Score**	**Number of clinicians**
Always	12 (63%)
Frequently	7 (37%)
Infrequently	0
Never	0

**Table 10:** Typical responses to the question *Can you envisage contexts where you would use the summaries?*

**Comment**
“In all clinical scenarios.”
“I think when people are in outpatient clinics it would be helpful to have a summary like this as it is time consuming to have to go through all the notes and you may miss things out. It is much easier to get a feel for time scale of events and to see what treatments/procedures the patient has had.”
“Patients who have received long treatment or have been looked after by the medical team for long periods of time.”
“Ward rounds and clerking of patients in A and E where quick summaries of salient points are needed and a clear concise picture of treatment and presentation.”
“Think they would be very useful. Can think of quite a few examples. When looking through long and complicated histories from patients were you are seeing/treating/managing them for the first time (say in A&E). Managing patients on the ward (who have presented with other problems) and you want to get a succinct history without having to look through pages and pages of old notes. When trying to get an idea of the story of a patient (i.e., how long their previous treatment has been, what they have previously tried, etc.). Gives an idea of what date to look for other documentation (i.e., referral letters, blood results, pathology requests, etc.) so that can find that information quicker. When referring patients, can copy the summaries to give the referree an idea of past medical history.”
“Yes, when answering quickfire questions on ward rounds, concerning aspects of patient history. Also, serve as very quick summary re- minders of the hisory of complex patient histories. Helpful to on call teams when reviewing patients.”
“When referring patients, when presenting patients, when considering further treatment to see what was successful in the past. etc., when reminding yourself of a patients history before seeing them in clinic, etc.”

**Table 11:** Typical responses to the question *What things didn’t you like about the summaries?*

**Comment**
“Not enough detail.”
“No indication if hypercalcaemia or anaemia is symptomatic, or if pain is controlled – generally if the patient is well and carrying out normal activities or not.”
“Concern that you’re not getting all the info!”
“Preferred the longitudinal summary, with more detail about presenting complaint would be more useful.”
“A bit too short – a bit more detail required.”
“I liked everything.”
